# A Hybrid Model with Quantum Feature Map Based on CNN and Vision Transformer for Clinical Support in Diagnosis of Acute Appendicitis

**DOI:** 10.3390/biomedicines14010183

**Published:** 2026-01-14

**Authors:** Zeki Ogut, Mucahit Karaduman, Pinar Gundogan Bozdag, Mehmet Karakose, Muhammed Yildirim

**Affiliations:** 1 Department of Surgery, Elazig Fethi Sekin City Hospital, Elazig 23300, Türkiye; drzeki44@gmail.com; 2 Department of Software Engineering, Malatya Turgut Ozal University, Malatya 44210, Türkiye; mucahit.karaduman@ozal.edu.tr; 3 Department of Radiology, Elazig Fethi Sekin City Hospital, Elazig 23300, Türkiye; pbozdag23@gmail.com; 4 Department of Computer Engineering, Firat University, Elazig 23119, Türkiye; mkarakose@firat.edu.tr; 5 Department of Computer Engineering, Malatya Turgut Ozal University, Malatya 44210, Türkiye

**Keywords:** artificial intelligence, quantum, appendicitis, CNN, ViT, 6G

## Abstract

**Background/Objectives**: Rapid and accurate diagnosis of acute appendicitis is crucial for patient health and management, and the diagnostic process can be prolonged due to varying clinical symptoms and limitations of diagnostic tools. This study aims to shorten the timeframe for these vital processes and increase accuracy by developing a quantum-inspired hybrid model to identify appendicitis types. **Methods**: The developed model initially selects the two most performing architectures using four convolutional neural networks (CNNs) and two Transformers (ViTs). Feature extraction is then performed from these architectures. Phase-based trigonometric embedding, low-order interactions, and norm-preserving principles are used to generate a Quantum Feature Map (QFM) from these extracted features. The generated feature map is then passed to the Multiple Head Attention (MHA) layer after undergoing Hadamard fusion. At the end of this stage, classification is performed using a multilayer perceptron (MLP) with a ReLU activation function, which allows for the identification of acute appendicitis types. The developed quantum-inspired hybrid model is also compared with six different CNN and ViT architectures recognized in the literature. **Results**: The proposed quantum-inspired hybrid model outperformed the other models used in the study for acute appendicitis detection. The accuracy achieved in the proposed model was 97.96%. **Conclusions**: While the performance metrics obtained from the quantum-inspired model will form the basis of deep learning architectures for quantum technologies in the future, it is thought that if 6G technology is used in medical remote interventions, it will form the basis for real-time medical interventions by taking advantage of quantum speed.

## 1. Introduction

Acute appendicitis is one of the most common emergency surgical indications worldwide [1]. Its prevalence in all age groups, its ability to affect large populations, and the fact that it leads to hundreds of thousands of appendectomies each year demonstrate the importance of this disease both clinically and epidemiologically [2]. Clinical evaluation for the diagnosis of acute appendicitis is sometimes inadequate due to symptomatic similarities with other intra-abdominal diseases. Delays in diagnosis can result in perforation, abscess, peritonitis, sepsis, and even mortality [3]. Furthermore, unnecessary surgery can be performed on patients with misdiagnosis. Variable initial symptoms, differences in laboratory parameters, and the inability to perform radiological evaluation due to limited resources all affect the sensitivity and specificity of the diagnosis [4]. Laboratory tests alone are not definitive diagnostic tools [5]. Failure to perform appropriate radiological evaluation, or the lack or absence of specialists, can lead to inaccurate or delayed diagnoses [6]. Therefore, the need for rapid, easily accessible, low-cost, and objective methods continues.

Accurate and rapid diagnosis of acute abdominal pathologies is vital for patient management. Artificial intelligence (AI)-based clinical decision support systems are increasingly being used in situations requiring urgent treatment [7]. These systems integrate demographic, clinical, laboratory, and radiological data, identify diseases through learned models, and can support clinicians by inferring disease stage. AI algorithms can improve diagnostic accuracy by capturing patterns that humans may overlook in imaging studies such as ultrasonography (USG), computed tomography (CT), or magnetic resonance imaging (MRI) [8]. Although limited in the literature, multimodal AI models that combine blood tests and abdominal CT images in the diagnosis of acute appendicitis are a developing field [9]. Most studies on this common clinical condition in the general population have used only laboratory data or radiological parameters [10,11]. This demonstrates that decision support systems in the diagnosis of acute appendicitis fall short of a sufficiently holistic approach.

In addition to diagnostic scoring methods used to diagnose acute appendicitis, systemic inflammatory markers are also used as biomarkers [12]. Indices derived from hemogram parameters, such as the ratio of total leukocyte count to lymphocyte or neutrophil subfractions (WBC/lymphocyte, WBC/neutrophil), the neutrophil/lymphocyte ratio (NLR), the lymphocyte/platelet ratio (PNR), and the platelet/lymphocyte ratio (PLR), are used to assess the systemic manifestations of the inflammatory response. Many studies have found statistically significant differences in these hematological ratios in cases of advanced, complicated appendicitis [13,14]. These parameters are combined with existing diagnostic data rather than used individually. The use of multimodal systems utilizing clinical, laboratory, and imaging data will increase diagnostic accuracy. This method will facilitate faster diagnosis, reduce unnecessary appendectomies, and help clinicians make more accurate decisions. Artificial intelligence-based decision support systems that analyze complex patient data and derive conclusions can help plan personalized and effective treatment for acute appendicitis. This will reduce clinicians’ subjective interpretations, expedite the diagnostic process, and fill a significant gap, particularly in centers with limited diagnostic tools.

This study aims to develop a quantum-inspired hybrid model that can improve histopathological diagnostic accuracy by integrating abdominal CT images. The innovative aspects of the developed model and its contributions to the literature are listed below.

- A new dataset consisting of abdominal CT images and five different classes was created for appendicitis detection. We have not encountered a dataset as comprehensive as the one we created in the literature. This will contribute to the literature.
- A quantum-inspired model was developed to identify appendicitis types from abdominal CT images.
- A Quantum Feature Map (QFM) [15,16,17] was created in the proposed model, incorporating high-level features of the two selected architectures, phase-based trigonometric embedding, low-rank interaction, and norm conservation principles. A Hadamard token was then generated. The MHA layer was input, generating the output for the final stage of the model [18,19]. The high-dimensional and computationally intensive structure of this hybrid quantum-inspired model will lay the groundwork for quantum computers and 6G-supported real-time medical artificial intelligence systems that will become widespread in the future.
- The results of the quantum-inspired model were also compared with six different CNN and ViT architectures accepted in the literature. Among these models, the highest accuracy of 97.96% was achieved in the quantum-based hybrid model.

The remainder of the article presents the material and methods, experimental results, discussion, and conclusion sections.

## 2. Materials and Methods

### 2.1. Study Design and Data Collection

This study was designed as an observational analysis to retrospectively evaluate patients who underwent appendectomy for acute appendicitis at the General Surgery Clinic of Elazig Fethi Sekin City Hospital between August 2018 and 31 December 2024. Demographic and clinical data of these patients were examined. The study aimed to record demographic data (age and sex), preoperative diagnostic laboratory parameters (complete blood count, total bilirubin, and C-reactive protein), and histopathological results from electronic patient files in the Hospital Information Management System (HIMS). Hematological parameters such as NLR, PLR, PNR, WBC/lymphocyte count, and WBC/neutrophil values were calculated and recorded. Preoperative abdominal computed tomography images obtained during the diagnostic phase were retrieved from the Image Archiving and Communication System (PACS). Data from a total of 3176 patients were evaluated, and a representative abdominal CT image was selected from each patient to create the study dataset. All data were anonymized.

According to histopathological results, the cases were divided into the following 5 groups:

1. Appendectomies with a normal appendix;

2. Appendectomies with simple (catarrhal) appendicitis;

3. Appendectomies with localized complications;

4. Appendectomies with advanced complications;

5. Appendectomies with rare histopathological variants (such as parasitic, neoplastic, or granulomatous appendicitis).

Table 1 shows detailed histopathological features of the patients included in the study.

**Figure 1:**
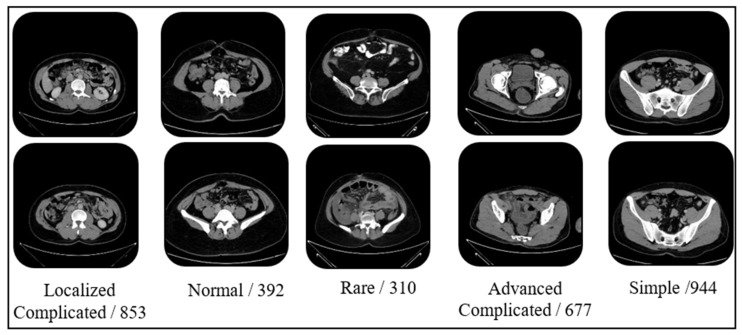
Sample images from the dataset.

Sample images and data numbers from the dataset are presented in Figure 1.

### 2.2. Inclusion and Exclusion Criteria

Inclusion criteria:

- Patients aged 18 years and older who underwent appendectomy between August 2018 and December 2024.
- Patients whose post-appendectomy histopathological evaluation results were available in the hospital electronic file.
- Patients whose laboratory test results were available within 24 h of surgery and whose preoperative abdominal CT images were recorded in the PACS system.

Exclusion criteria:

- Patients under the age of 18 who underwent appendectomy.
- Patients with missing histopathological evaluation or preoperative laboratory data.
- Patients with concurrent intra-abdominal malignancies or prior major abdominal surgery.
- Patients who underwent appendectomy during interval appendectomy or other surgical procedures.

### 2.3. Ethical Approval

Approval for the study was obtained from the Elazig Fethi Sekin City Hospital Non-Interventional Clinical Research Ethics Committee (Decision No: 2025/17-14, Date: 16 October 2025). The research was conducted in accordance with the ethical principles of the Declaration of Helsinki, and all data were anonymized in accordance with confidentiality principles.

### 2.4. Quantum-Inspired CNN and the ViT-Based Proposed Model

**Figure 2:**
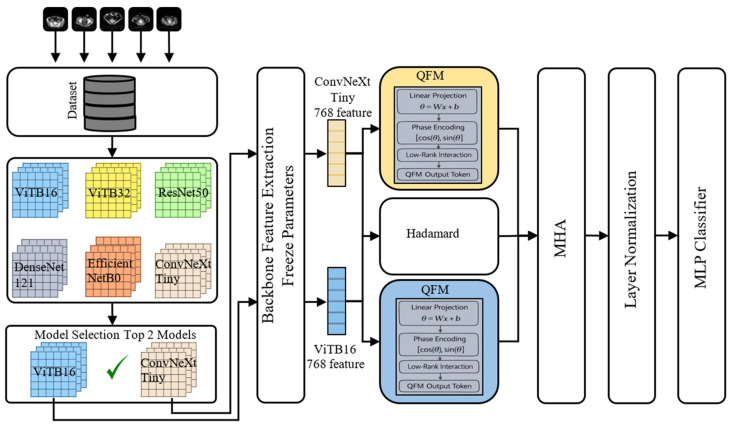
Proposed model flowchart.

In this study, we propose a deep learning-based model for multi-class classification of image data from patients who underwent appendectomy for acute appendicitis. The model first classifies the dataset using pre-trained deep learning architectures, then designs an architecture using the two best architectures as the base. Due to the importance of balancing precision and recall in medical datasets, the F1 score was used as the primary criterion instead of accuracy in model evaluation, and the final model selection was made based on this criterion. The flowchart of the proposed model is shown in Figure 2.

Figure 2 shows a performance comparison of four CNNs and two ViT architectures during the selection phase to identify the best models. The pre-trained architectures ViTB32, ViTB16, ConvNeXtTiny, ResNet50, EfficientNetB0, and DenseNet121 were chosen due to their different key features. ViTB16 was selected as a transformer because of its small patch size, and ViTB32 because of its large patch size and low computational cost. ConvNeXtTiny was chosen for its modern architecture and low computational cost, ResNet50 for its stability and reliability thanks to its residual connection structure, EfficientNetB0 for its high parameter efficiency, and DenseNet121 for its high feature reuse due to its dense connection structure. Based on experimental results, the two models demonstrating the best performance were selected and will be used as the backbone in the proposed architecture. Feature extraction is performed using the two selected architectures. The extracted features are combined by creating a QFM [15,16,17,20]. A fusion architecture is then created by adding a layer containing MHA [18,19].

In the data preprocessing step, all images are scaled to a size usable by the available architectures. Then, using Equations (1) and (2), the channel-based mean and standard deviation are calculated from all data.

(1) $$\mu_{c}=\frac{1}{NHW}\overset{N}{\underset{n=1}{\sum }}\overset{H}{\underset{h=1}{\sum }}\overset{W}{\underset{w=1}{\sum }}x_{n,c,h,w}\;\;,\;c\;\in \left\{1,\;2,\;3\right\}$$

(2) $$\sigma_{c}=\sqrt{\frac{1}{NHW}\overset{N}{\underset{n=1}{\sum }}\overset{H}{\underset{h=1}{\sum }}\overset{W}{\underset{w=1}{\sum }}{\left(x_{n,c,h,w}-\mu_{c}\right)}^{2}},\;c\;\in \left\{1,\;2,\;3\right\}\;$$

Here, $\mu_{c}$ represents the channel mean, $\sigma_{c}$ is the channel standard deviation, $\left(h,w\right)$ represents the spatial location, x represents the pixel value, *c* represents the channels in the image, *N* is the total number of images, *H* is the vertical pixel dimension of the image, and *W* is the horizontal pixel dimension of the image. Using the calculated channel-based mean and standard deviation, each image is normalized pixel-by-pixel as in Equation (3).

(3) $${\tilde{x}}_{n,c,h,w}=\frac{x_{n,c,h,w}-\mu_{c}}{\sigma_{c}}$$

At this stage, the images are split into training and test sets. The data is split into 80% for training and 20% for testing, with 10% of the training data allocated to validation at each epoch.

Six different architectures were selected as pre-trained architectures. Four of these architectures are CNN architectures: ResNet50 [21], DenseNet121 [22], EfficientNetB0 [23], and ConvNeXtTiny [24], while two are transformer architectures: ViTB16 [25] and ViTB32 [26]. The pre-trained architectures were fine-tuned on the dataset, and their classification performance was compared. All architectures were trained with the cross-entropy loss defined in Equation (4).

(4) $$L_{CE}=-\frac{1}{N_{b}}\overset{N_{b}}{\underset{n=1}{\sum }}\log p_{n,y_{n}}$$

$N_{b}$ represents the minimum batch size, $y_{n}$ is the true class label, $p_{n,y_{n}}$ is the probability of belonging to the correct class in the Softmax output, and $L_{CE}$ is the average cross-entropy loss.

For each architecture, the macro F1 score is used for early stopping. Accuracy, precision, recall, and F1 score are used for performance comparison.

After comparing based on the F1 score, the best-performing architectures are selected as Backbone A and Backbone B. The classifier layers of these architectures are removed and configured to serve as fixed feature extractors. The resulting features are used to generate an interaction vector using the Hadamard [20,27,28]. For dimension alignment, the smallest dimension is used according to Equation (5).

(5) $$d_{H}=\min\left(d_{A},d_{B}\right)$$

In Equation (5), $d_{A}$ is the feature vector dimension produced by backbone architecture A, $d_{B}$ is the feature vector dimension produced by backbone architecture B, and $d_{H}$ is the dimension of the Hadamard interaction vector. The two architectures are cropped to the dimension of the Hadamard interaction vector [20,27,28]. The new vectors, with their adjusted dimensions, are multiplied component-wise to create a third vector, as in Equation (6).

(6) $$z_{H}={\hat{z}}_{A}⊙{\hat{z}}_{B}$$

In Equation (6), ${\hat{z}}_{A}$ is the clipped vector of spine A, ${\hat{z}}_{B}$ is the clipped vector of spine B and $z_{H}$ is the newly created Hadamard vector. Quantum-inspired feature mapping is used to generate a QFM [15,16,17]. For each map, the phase projection is created by combining the low-rank interaction values and L^2^-normalization components [29]. Equations (7) and (8) are used to generate the phase projection and the trigonometric embedding.

(7) $$\theta =W_{z}+b\;\in \;{\mathbb{R}}^{P}$$

(8) $$c=\cos\left(\theta \right),s=\sin\left(\theta \right),c,s\;\in \;{\mathbb{R}}^{P}$$

Here, z is the feature vector extracted from the backbone architecture, W is the learnable projection matrix, b is the bias value, θ is the phase vector, c is the cosine transform, and s is the sine transform. To calculate the low-rank interaction vector, sin and cos calculations are performed, and the result is obtained via component-based multiplications. This process mimics the weak-interaction principle in quantum systems by creating complex feature relationships at low parameter cost. Equations (9) and (10) illustrate this calculation.

(9) $$h_{1}=\cos\left(\theta U\right),\;\;\;h_{2}=\sin\left(z^{T}V\right)$$

(10) $$h=h_{1}⊙h_{2}$$

Here, $h_{1}$ represents the trigonometric interaction component in the phase domain, $h_{2}$ represents the trigonometric interaction component in the input domain, and h represents the low-rank interaction vector. The extended feature representation is defined as $\tilde{f}=\left[c;\;s;\;h\right]$. Equation (11) is used to project it through the linear layer and transform it into a more meaningful feature vector.

(11) $$f=W_{0}\;\tilde{f}+b_{0}$$

In Equation (11), $W_{0}$ is the projection matrix that reduces the combined vector to *P* dimensions, $b_{0}$ is the output layer bias term, and f is the feature vector obtained from the final projection. In the final stage, by scaling the output using the L2 norm, a stability similar to the norm conservation observed in quantum states is achieved, and the distribution of features is appropriately adjusted during training. Equation (12) is used for the calculation.

(12) $$\psi =\frac{f}{{\left\|f\right\|}_{2}+\varepsilon }$$

In Equation (12), ψ is the output of the QFM, ε is a small positive constant for numerical stability, and ${\left\|f\right\|}_{2}$ is the L2 norm. Thanks to this structure, QFM combines complementary components that enhance classical deep learning features, such as phase, trigonometric embedding, and low-rank interactions, to produce a more discriminative and more easily learned representation for the model. The three outputs obtained from the QFM modules are the token for spine A, the token for spine B, and the Hadamard interaction token. Each image is converted into a short sequence of three elements: one token per image. Thus, each image is represented by a sequence of three pieces of information. The sequence of three elements is expressed in Equation (13).

(13) $$T=\left[\begin{array}{c} t_{A}^{T}\\ t_{B}^{T}\\ t_{H}^{T} \end{array}\right]\;\;\;\;\;\in \;{\mathbb{R}}^{3xP}$$

The sequence of three tokens in Equation (13) is then passed to the MHA mechanism. In this mechanism, a separate query, key, and value transformation is applied for each head. The learnable projection matrices are $W_{h}^{Q}$, $W_{h}^{K}$, and $W_{h}^{V}$, and MHA calculations are performed using Equation (14).

(14) $$Attention\left(Q,K,V\right)=softmax\left(\frac{QK^{T}}{\sqrt{d_{k}}}\;\;\right)V$$

In Equation (14), Q is the query matrix, K is the key matrix, V is the value matrix, and $Attention\left(Q,K,V\right)$ is the resulting attention output.

The MHA layer added to the proposed model is integrated to learn deep contextual relationships in images. In addition to the local features captured by pre-trained architectures, MHA can also capture contextual features. The MHA structure incorporated into the model re-represents the features from the Hadamard output using three fundamental matrices: Q, K, and V, defined in Equation (14). Of these matrices, Q is used to query the relationship of a feature at a specific location to other locations. K is the reference matrix containing the features of each location and helps determine which locations are important for attention. The V matrix represents the information at the location and is used to generate the output by weighting it according to attention scores. The multi-head version is as given in Equation (15). Here, multiple query–key–value groups are calculated in parallel, and each head learns different relationships and feature subspaces [18,19].

(15) $$MHA\left(Q,K,V\right)=\left[head_{1};\ldots ;head_{h}\right]W^{O}$$

In Equation (15), h is the number of attention heads used, $head_{i}$ is the output of the ith attention head, and $W^{O}$ is the output projection matrix. The representation vector obtained from the model’s fusion stage was fed into a multilayer perceptron (MLP) for classification. This classifier consists of a hidden layer and a ReLU activation structure. A dropout layer was used to reduce overfitting, and in the final layer, a transformation was applied to generate probability values for each class. The features enhanced with the QFM and MHA structures were converted into the final decision vector using the MLP, and class prediction was performed.

## 3. Results

In this study, a quantum-inspired model was developed to manage acute appendicitis. Since the study used an original dataset, the performance metrics of the developed quantum-based hybrid model were compared with six different CNN and ViT architectures reported in the literature. The models used for acute appendicitis detection in the study are ViTB32, ViTB16, ConvNeXtTiny, ResNet50, EfficientNetB0, and DenseNet121. These models were trained using AdamW on 224 × 224 normalized images with ImageNet weights for 10 epochs, and the outputs of the top two models were fed into the QFM- and MHA-based fusion architecture. The images are sized as 224 × 224 because this is the input image size for the pre-trained architectures. The AdamW optimization algorithm was chosen and used as a standard in all architectures because it provides effective regularization by separating weight decay from gradient updating, reduces the risk of overfitting, and aims to achieve good generalization performance. The number of features extracted from each of the two architectures selected as the backbone is 768. The QFM modules consist of a phase embedding dimension of 256, a low-rank interaction dimension of 32, and L2 normalization components. The MHA part uses a self-attention mechanism titled 4. All experiments were run on an NVIDIA Tesla T4 GPU with a batch size of 32, a learning rate of 1 × 10^−4^, and a cosine annealing learning rate schedule. Various evaluation metrics, including accuracy, precision, recall, and F1 score, were used to compare the model’s performance.

**Figure 3:**
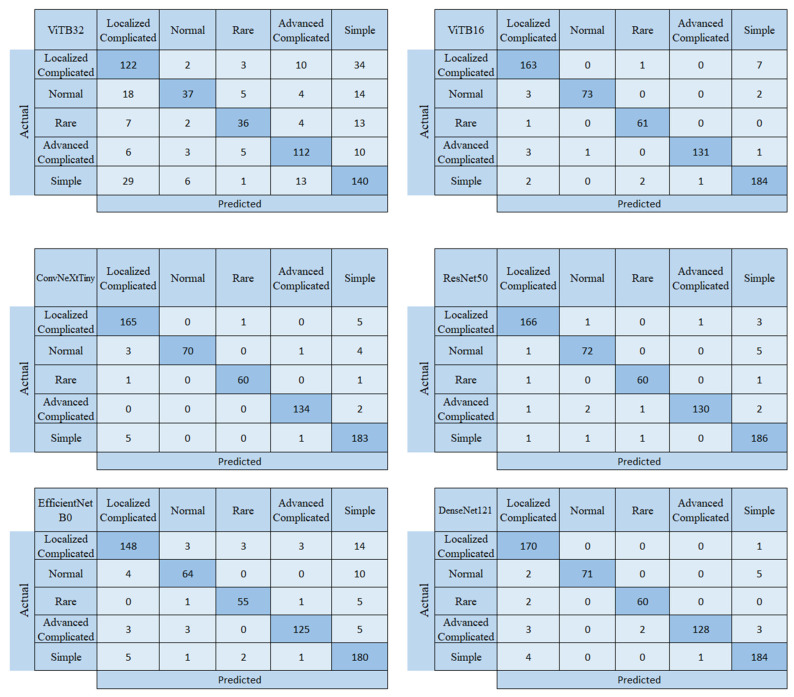
Confusion matrices of the CNN and ViT architectures.

In this section, we first present the confusion matrices and performance evaluation metrics obtained from the CNN and ViT architectures. We then present the confusion matrix and performance metrics for the proposed quantum-based model. The confusion matrices for the CNN- and ViT-based models are shown in Figure 3.

**Figure 4:**
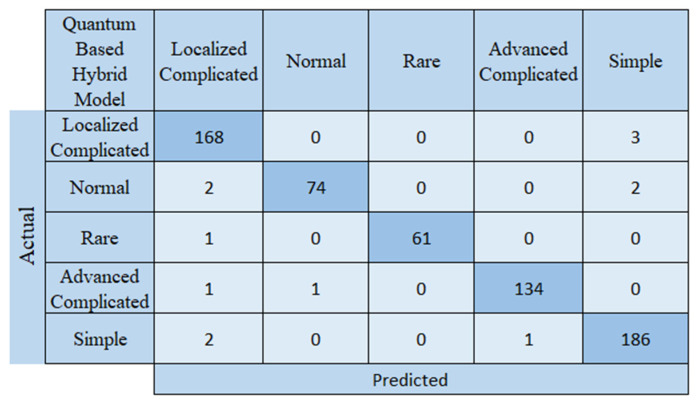
Confusion matrix of the proposed model.

When the confusion matrices from the study on acute appendicitis detection are examined, it is seen that the most successful model is ResNet50 and the least successful is ViTB32. However, when we look at the F1 score, which is the most important factor in this study and which we used in model selection, it is seen that the best performance belongs to the VITB16 architecture, and secondly to the ConvNeXtTiny architecture. Therefore, these two models were chosen as the backbone. The ResNet50 architecture correctly predicted 614 of the 636 test images and incorrectly predicted 22. While the ResNet50 architecture correctly predicted 166 of the 171 test images in the localized complicated class, it incorrectly predicted 1 test image as normal, 1 as advanced complicated, and 3 as simple appendicitis. In the normal class, it correctly predicted 72 of the 78 test images and incorrectly predicted 6. In the rare class, it predicted 60 correctly and 2 incorrectly; in the advanced complicated class, 130 correctly and 5 incorrectly; and in the simple class, 186 correctly and 3 incorrectly. The other most successful model after ResNet50 was DenseNet121. The DenseNet121 model incorrectly predicted 23 of the test images; ViTB16 and ConvNeXtTiny incorrectly predicted 24; EfficientNetB0 incorrectly predicted 64; and ViTB32 incorrectly predicted 189. The confusion matrix for the quantum-based hybrid model developed for the appendicitis method, which produced the best results, is shown in Figure 4.

An examination of Figure 4 shows that the quantum-based hybrid model incorrectly predicted 13 of the 636 test images, while correctly predicting 623. These results, when compared with the other models used in the study, demonstrate that the proposed model is more successful. Of the pre-trained models, ResNet50 appears to be the most successful. The proposed quantum-based hybrid model incorrectly predicted 13 test images, while ResNet50 incorrectly predicted 22. The other models had more errors. The performance metrics of the proposed quantum-based model for appendicitis management are presented in Table 2.

An examination of Table 2 reveals that the rare class has the highest precision. The value obtained in this class is 100%. The class in which the model performs least well is the localized complicated class. The precision value obtained in this class is 96.55%. Table 3 presents the performance metrics obtained when the proposed model is compared to other accepted models in the literature.

An examination of Table 3 reveals that the proposed quantum-inspired model outperforms the other models used in the study. The proposed model has better metric values than all the models used in the study. The proposed model achieved 97.96% accuracy and 97.96% F1 score.

## 4. Discussion

The quantum-inspired model developed in this study can detect acute appendicitis and also distinguish the degree of complication. An examination of the proposed model’s confusion matrices and performance evaluation metrics clearly demonstrates its success. The proposed model successfully distinguished between mildly complicated and severely complicated appendicitis. The distinction between these two classes can lead to disagreements among experts. The high detection rate in the severely complicated class will enable early surgical planning, a crucial step in preventing sepsis. The proposed quantum-inspired model will enable a decision on a conservative or surgical approach after the diagnosis is clarified. The proposed model will provide an objective assessment, independent of the surgeon’s experience.

Acute appendicitis is one of the most frequently encountered abdominal pathologies worldwide that requires urgent surgical intervention. It is estimated that approximately 7–8 million people worldwide undergo surgery for acute appendicitis each year [1,30,31]. One of the main reasons for the high number of surgical procedures performed for acute appendicitis is the inability to detect it at an early stage. The lack of adequate laboratory and imaging techniques is a significant factor in appendicitis, where early detection is crucial [32]. Furthermore, nonspecific laboratory and imaging results are another factor that complicates the diagnostic process [33]. Failure to diagnose at an early stage can lead to morbidity and mortality, especially in elderly patients [34]. Furthermore, late diagnosis in patients with comorbidities significantly affects outcomes. Therefore, early detection of acute appendicitis using computer-aided systems is of great importance. Early diagnosis enables more accurate, faster treatment planning for patients. This will shorten hospital stays and significantly reduce mortality rates. Early diagnosis with computer-aided systems will also significantly reduce hospital costs.

Complicated appendicitis has higher complication rates than simple appendicitis. Patients with complicated appendicitis have longer hospital stays. However, complications such as surgical site infection, intra-abdominal abscess, ileus, and sepsis are more common in these patients than in other appendicitis groups [35]. According to a study by Alotaibi et al., hospital stays can be two to six times longer in patients with complicated appendicitis. These patients also have a higher 30-day readmission rate [36]. In this group, where the risk of complications is increased, treatments should be individualized using artificial intelligence-supported systems. As seen in that study, the simple appendicitis group and the mildly complicated appendicitis group may exhibit similar clinical and laboratory findings. The accuracy rates achieved by the model in these two groups were 98.4% and 98.2%, respectively, and confusion matrix analysis revealed a small number of cross-classifications between the simple and mildly complicated groups (3 cases in each group). The lack of a sharp difference between them at the histopathological level also approximates the symptoms and findings of this group [37]. Therefore, AI-assisted classification is important in distinguishing these two groups. Uncommon variants of appendicitis, such as neuroendocrine tumors, mucinous neoplasms, endometriosis, or metastatic involvement, generally do not present with classic symptoms [38]. In that study, the number of cases in this group is limited. This relates to the rarity of these diagnoses in this group in the general population. The model we developed distinguished rare appendicitis variants with 98.4% accuracy (61/62). Because these cases are more challenging to diagnose, the decision-support insights derived from AI models become even more critical.

Artificial intelligence models can provide faster and more accurate diagnostic results than traditional clinical scoring systems (Alvarado, AIR, and RIPASA) [39]. This is because future models will be able to evaluate additional data alongside these scores. In a systematic review of 6 artificial intelligence studies, Chan et al. reported that machine learning (ML) models demonstrated higher diagnostic accuracy than traditional scoring systems, such as the Alvarado score [40], for the diagnosis of acute appendicitis. Many models have been developed, especially in recent years [41,42]. It is believed that these developments will enable even more successful results in the future. We believe that the proposed quantum-inspired model could play a significant role in preventing unnecessary surgeries and reducing complications in children, the elderly, and atypical cases. Furthermore, our proposed model will shorten the time to early diagnosis and enable early treatment initiation. The use of single-center data, the small number of patients in some groups, and the lack of analysis of physical examination data are among the limitations of our study. Model performance has not been tested on patient-based data partitioning and external datasets from different imaging devices. Therefore, how the results would change under different validation scenarios has not been evaluated within the scope of this study. In our future work, our aim is to make real-time applications with multi-center data.

## 5. Conclusions

In this study, a five-class dataset was created by two experts in the field for the management of acute appendicitis. Acute appendicitis was graded from this dataset. A quantum-inspired hybrid model based on CNN and ViT architectures was then developed for the automatic early detection of acute appendicitis. The developed model was designed with the understanding that it could guide future AI-based models for quantum technologies and, if 6G technology is used for remote medical interventions, could form the basis for real-time medical interventions by leveraging quantum speed. The developed model achieved an appendicitis grade with 97.96% accuracy. This value demonstrates that the developed model will reduce specialists’ workload and can be used for preliminary diagnosis in settings without specialists. Furthermore, the developed quantum-inspired hybrid model has the potential to prevent traditional errors that specialists may make.

## Figures and Tables

**Table 1:** Classification criteria based on the histopathological results of patients in the groups.

Group Name	Histopathological Diagnosis/Findings
Normal appendix	Appendix tissue with no signs of acute inflammation in histopathological examination.
Simple (catarrhal) appendicitis	Histopathologically, catarrhal-type inflammation with superficial neutrophil infiltration, hyperemia, and edema in the mucosa, without perforation or abscess.
Localized complicated appendicitis	Transmural inflammation, necrosis, small foci of abscess, or limited perforation.
Advanced complicated appendicitis	Appendectomies with full-thickness necrosis, periappendicular abscess, fibrinous peritonitis, large perforation, and gangrenous inflammation findings
Rare histopathological variants	It consists of cases with clinical or radiological findings similar to those of acute appendicitis but with histopathologically distinct etiologies. This group includes low-grade appendiceal mucinous neoplasms, mucinous adenocarcinomas, mucoceles, well-differentiated neuroendocrine tumors, endometriosis, parasitic appendicitis (e.g., Enterobius vermicularis), sessile serrated lesions, villous or serrated adenomas, metastatic tumors, and other rare malignancies.

**Table 2:** Class-based performance metrics of proposed models.

Class	Precision % (Test)	Recall % (Test)	F1 Score % (Test)
Localized Complicated	96.55	98.25	97.39
Normal	98.67	94.87	96.73
Rare	100.00	98.39	99.19
Advanced Complicated	99.26	98.53	98.89
Simple	97.38	98.41	97.89

**Table 3:** Performance metrics comparison for all models.

Models	Weighted F1 Score % (Test)	Accuracy Rate % (Test)	Error Rate % (Test)	Weighted Precision % (Test)	Weighted Recall % (Test)	Train Avg Epoch Time (Sec)	Infer per Image (ms)
VITB32	68.30	70.28	29.72	71.54	66.66	28.02	7.58
EfficientNetB0	89.65	89.94	10.06	90.66	88.89	50.72	14.67
ResNet50	96.28	96.54	3.46	96.56	96.03	33.52	7.62
DenseNet121	96.32	96.39	3.61	97.05	95.74	30.40	7.96
ConvNeXtTiny	96.33	96.23	3.77	97.11	95.67	62.87	17.04
VITB16	96.35	96.23	3.77	96.56	96.20	33.93	9.02
Concat+MLP (ConvNeXtTiny+ VITB16)	96.60	96.38	3.62	97.25	96.38	28.88	8.33
Quantum Inspired Proposed Model	97.96	97.96	2.04	97.97	97.96	43.75	10.32

## Data Availability

Data used in this research are available upon request from the corresponding author.
